# Delivery of ENaC siRNA to epithelial cells mediated by a targeted nanocomplex: a therapeutic strategy for cystic fibrosis

**DOI:** 10.1038/s41598-017-00662-2

**Published:** 2017-04-06

**Authors:** Maria D. I. Manunta, Aristides D. Tagalakis, Martin Attwood, Ahmad M. Aldossary, Josephine L. Barnes, Mustafa M. Munye, Alexander Weng, Robin J McAnulty, Stephen L. Hart

**Affiliations:** 1 Experimental and Personalised Medicine Section, UCL Great Ormond Street Institute of Child Health, London, UK; 2 UCL Respiratory Centre for Inflammation and Tissue Repair, University College London, London, UK; 3 Centre for Cellular and Molecular Physiology, University of Oxford, Oxford, UK; 4 Institute of Pharmacy, Freie Universität Berlin, Berlin, Germany

## Abstract

The inhibition of ENaC may have therapeutic potential in CF airways by reducing sodium hyperabsorption, restoring lung epithelial surface fluid levels, airway hydration and mucociliary function. The challenge has been to deliver siRNA to the lung with sufficient efficacy for a sustained therapeutic effect. We have developed a self-assembling nanocomplex formulation for siRNA delivery to the airways that consists of a liposome (DOTMA/DOPE; L), an epithelial targeting peptide (P) and siRNA (R). LPR formulations were assessed for their ability to silence expression of the transcript of the gene encoding the α-subunit of the sodium channel ENaC in cell lines and primary epithelial cells, in submerged cultures or grown in air-liquid interface conditions. LPRs, containing 50 nM or 100 nM siRNA, showed high levels of silencing, particularly in primary airway epithelial cells. When nebulised these nanocomplexes still retained their biophysical properties and transfection efficiencies. The silencing ability was determined at protein level by confocal microscopy and western blotting. *In vivo* data demonstrated that these nanoparticles had the ability to silence expression of the α-ENaC subunit gene. In conclusion, these findings show that LPRs can modulate the activity of ENaC and this approach might be promising as co-adjuvant therapy for cystic fibrosis.

## Introduction

Cystic fibrosis (CF) is the most common autosomal recessive inherited disorder, affecting function of the exocrine epithelium. The first mutation in the cystic fibrosis transmembrane regulator (CFTR) gene was described in 1989^1, 2^. The disease is characterized by ionic imbalance that causes reduced volume of surface liquid in the airways, mucus dehydration and reduced mucus clearance. The increased activity of the epithelial Na^+^ channel (ENaC), associated at least partially with a faulty CFTR, contributes to the exacerbation of CF disease^3^. The amiloride-sensitive ENaC is in fact one of the key players in salt and water reabsorption in the epithelium, including the respiratory tract, where it maintains Na^+^ and water homeostasis and hence contributes to the maintenance of the correct volume of airway surface liquid in the human airways. In CF, dehydration of respiratory secretions and impaired mucociliary clearance, caused by the hyperabsorption of Na^+^ through ENaC in the absence of Cl^−^ secretion, heavily contributes to the lung pathology and respiratory insufficiency. The ionic imbalance results in the thick, sticky mucus secretions, flourishing of bacterial infections and, in the long term, in the progressive decline of lung function that is one of the major causes of CF morbidity.

The non-voltage gated sodium channel ENaC, belonging to the degenerin superfamily, consists of three main subunits, α-, β- and γ- formed of 669, 640, and 649 amino acids, respectively^4, 5^. The channel is believed to have a heterotrimeric structure and the three subunits appear to be essential for the assembly of functional channels on the cell membrane^6, 7^. ENaC biodistribution is quite variable among different cells and tissues^8^. A fourth subunit, δ-ENaC, has also been found in several human tissues, which, together with the β- and γ-subunits, can form a functional ion channel^9, 10^. However, the expression of the α-ENaC subunit appears to be essential for full channel function, while residual channel activity can be measured in the absence of the two others (β and γ)^11^. ENaC dysregulation due to a genetic mutation of the subunits has been linked with the pathogenesis of Liddle’s syndrome and salt-sensitive hypertension (pseudoaldosteronism)^12^. There is increasing evidence implicating ENaC dysregulation and hyperactivity in CF disease^3^.

The hypothesis of utilizing an ENaC-blocking molecule to facilitate restoration of the airway surface liquid (ASL) volume sufficiently to allow normal mucociliary clearance is of interest in the management of lung disease in CF patients^13^. The present study aimed to develop small interfering RNA (siRNA) nanoparticle formulations to be delivered to the airways. SiRNA is a feasible silencing strategy and we have shown that is possible to silence ENaC. Short interfering RNAs (siRNAs) are double-stranded RNAs 20–25 nucleotides long, which naturally regulate gene expression by degrading the messenger RNA^14^. Since the introduction of siRNA knockdown expression of specific genes in mammalian cells in 2001^14, 15^, there is a growing number of therapeutic applications to treat a wide range of diseases by gene silencing.

To deliver the siRNA against the α-subunit of ENaC, we have developed nanoparticle formulations (LPRs) consisting of a cationic liposome (DOTMA/DOPE), a peptide with a targeting motif and siRNA. The targeting moieties SERSMNF in peptide E and YGLPHKF in peptide Y have been identified by biopanning a phage peptide library^16^. The peptide motif Y displays close similarity to a targeting protein expressed by the intracellular pathogen *Legionella pneumophila* while peptide E closely resembles the receptor binding proteins of two intracellular pathogens, rhinovirus and *Listeria monocytogenes* ^16–18^ and both have been shown to provide better targeting specificity and efficiently mediate delivery to airway cells^19–21^. LPRs were evaluated for their transfection efficiency in silencing the α-ENaC gene *in vitro* and *in vivo*. Biophysical characteristics including size, charge and shape were determined before and after nebulisation to assess their stability to shear forces. The current study allowed us to understand in more detail the potential of LPRs as tools for siRNA delivery to the airways and to develop them further as promising therapeutic applications for CF.

## Results

### Targeted silencing of ENaC in cell lines transfected with LPR detected by qRT-PCR

**Figure 1:**
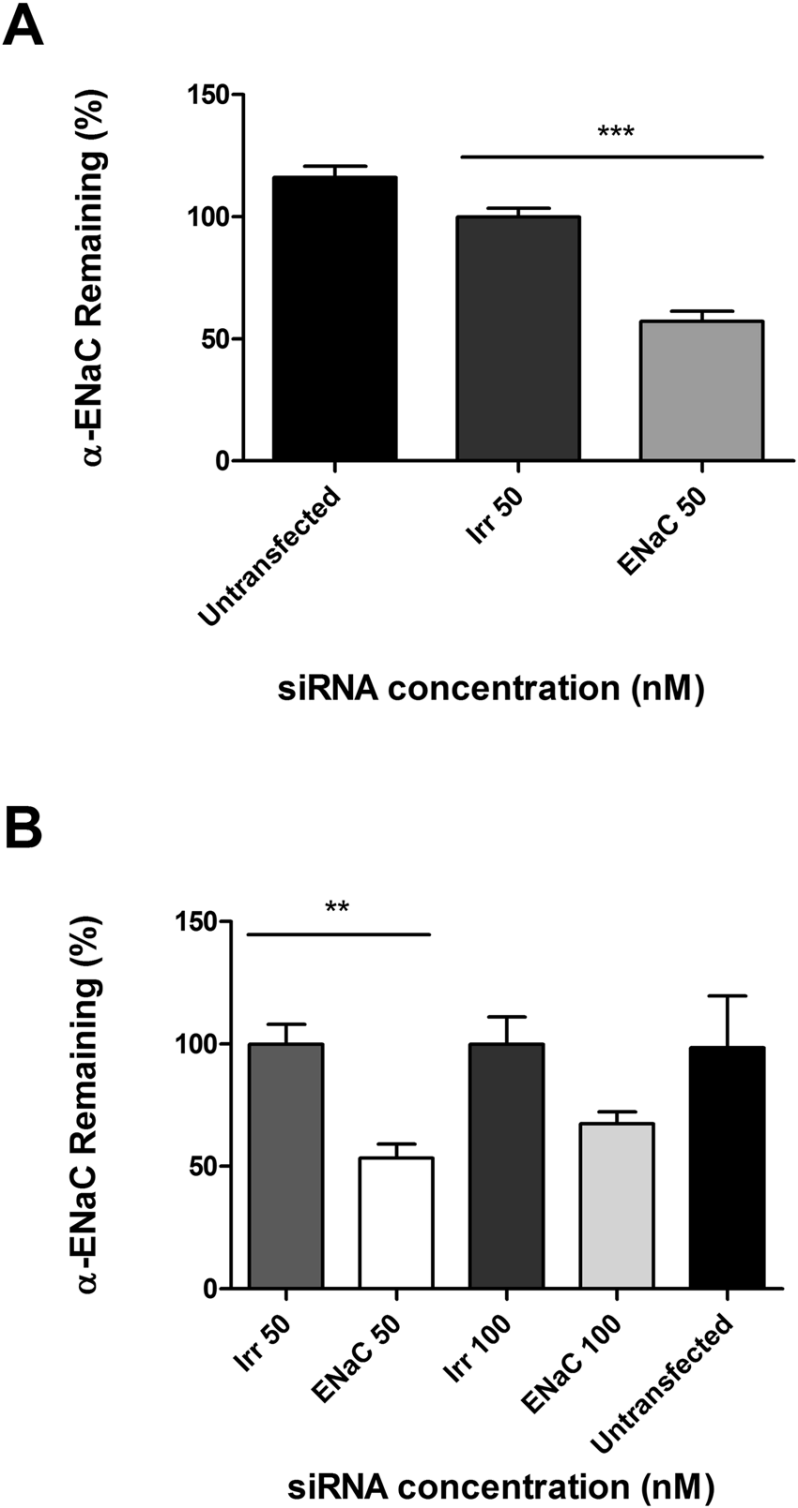
Silencing efficiency of cell lines transfected with LPRs containing peptide E. (**A**) In independent experiments, using 50 nM siRNA, the level of expression of α-ENaC gene in 16HBE14o- was of 57.2% in respect to GAPDH housekeeping gene (n = 7). Statistically significant difference was determined as ***P ≤ 0.001. (**B**) In A549 cells the remaining level of ENaC, expressed as percentage of non-targeting/irrelevant siRNA, was 53.4% at 50 nM, with a statistical difference of **P ≤ 0.01, and 67.5% at 100 nM. The results show the means ± S.E.M. of triplicate experiments.

LPR nanocomplexes, formulated with peptide E (K_16_GACSERSMNFCG) and containing either targeting ENaC or non-targeting/irrelevant siRNA at 50 nM, were used to transfect 16HBE14o- epithelial cells. Following siRNA treatment, the level of silencing of the α-ENaC gene, determined by qRT-PCR was 42.8% (Fig. 1A), normalised to the GAPDH housekeeping gene.

The efficiency of gene silencing of LPR nanoparticles was further examined in another cell line (A549). It showed an average silencing of 46.6% at 50 nM and of 32.5% at 100 nM (Fig. 1B). The 100 nM concentration was shown to be more toxic (see below), which may explain the lower level of silencing in these cells compared to 50 nM.

### Transfection of primary normal and CF airway epithelial cells and air-liquid interface (ALI) cultures

**Figure 2:**
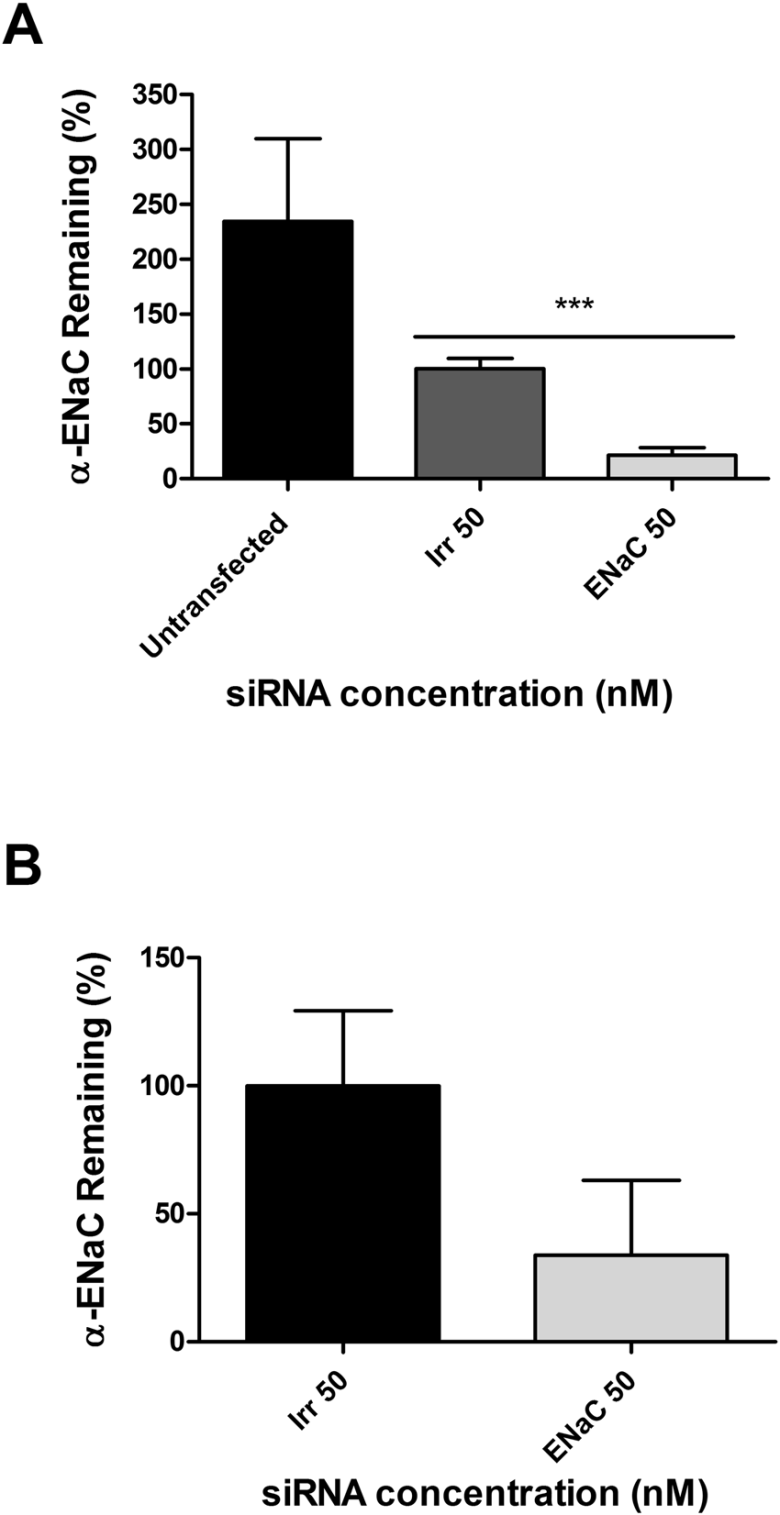
Knockdown of α-ENaC in primary cells transfected with peptide E nanoparticles. (**A**) The α-ENaC expression in NHBE submerged cultures (n = 5) treated with 50 nM siRNA was 21.5% relative to GAPDH housekeeping gene. The difference was statistically significant (*** P ≤ 0.001). (**B**) Air-liquid interface cultures of NHBE (n = 3) treated with LPRs containing 50 nM siRNA showed a remaining expression of 33.8% giving therefore a silencing of 66.2% relative to GAPDH housekeeping gene. The remaining expression of the α-ENaC gene is expressed as percentage relative to non-targeted/irrelevant siRNA-treated cells.

**Figure 3:**
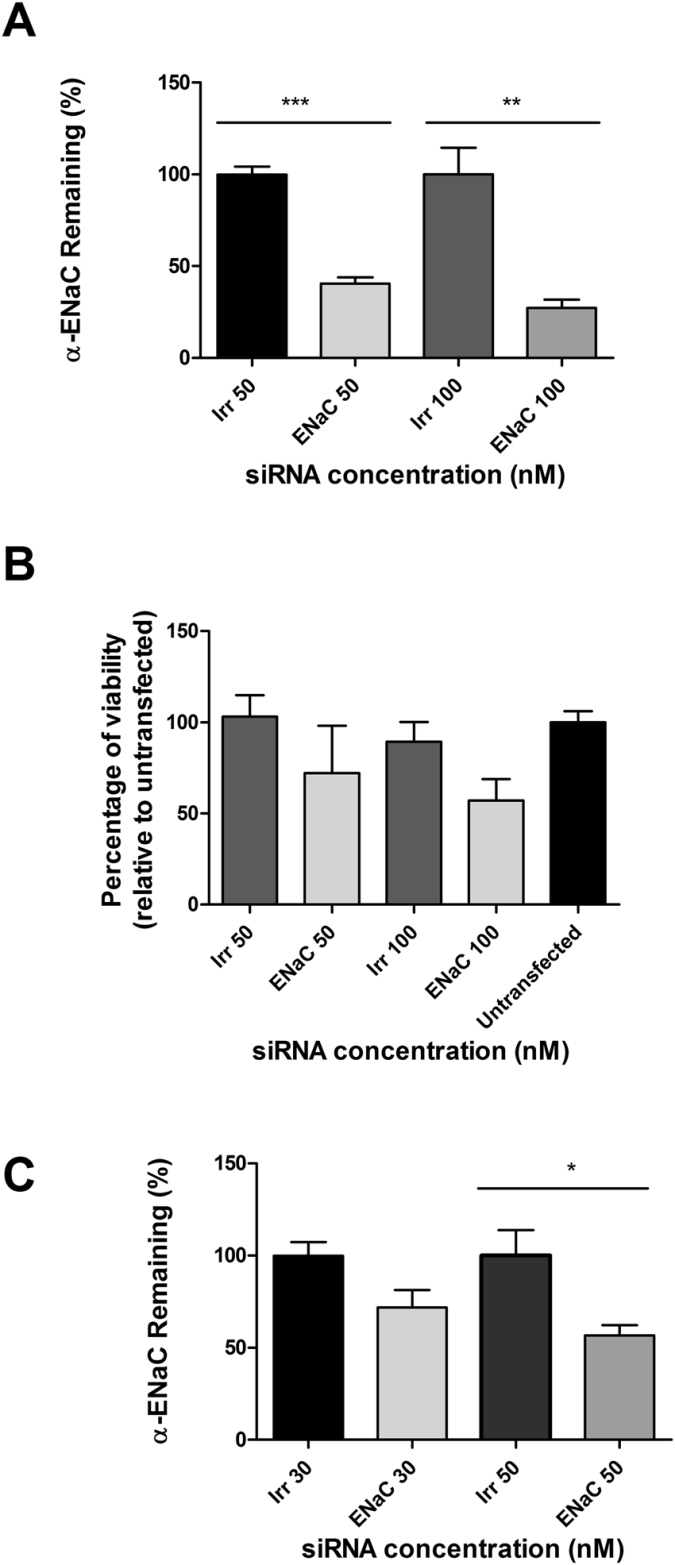
Primary CF epithelial cells with LPRs containing either 50 nM or 100 nM siRNA and peptide Y. (**A**) Percentage of expression of the α-ENaC mRNA after siRNA treatment in primary CF cells in submerged culture. The expression of α-ENaC was 40.5% at 50 nM and 27.2% at 100 nM, respectively. The difference was statistically significant (*** P ≤ 0.001) at 50 nM, whereas the significance (**P ≤ 0.01), was lower at 100 nM. The results show the means ± S.E.M. of triplicate experiments. (**B**) Viability (percentage) of primary CF cells following transfections with LPRs formulated with peptide Y compared to untransfected cells. The α-ENaC-siRNA treated cells showed a viability of 72% at 50 nM siRNA concentration and 57% at 100 nM. Cell survival was assessed using an LDH assay measuring the light emitted at 610 nm. Results represent mean values of triplicate repeats ± S.E.M. (**C**) Percentage of remaining expression of the α-ENaC gene transcript in primary CFBE cells grown on ALI in transwell plates. The expression of the transcript of the α-ENaC gene was 71.8% at 30 nM and 56.7% at 50 nM. The difference was statistically significant only at 50 nM (*P ≤ 0.05). qRT-PCR analysis was performed in triplicate on individual samples. The mRNA levels of the α-ENaC gene are expressed as percentage of irrelevant/non-targeted control siRNA.

We next compared the silencing efficiency of LPR nanoparticles formulated with peptide E at 50 nM siRNA in primary normal human bronchial epithelial cells (NHBE). The expression of α-ENaC in submerged NHBE showed a silencing efficiency of 78.5% (Fig. 2A). As expected, silencing was less efficient (66.2%) when the same cells were transfected in air-liquid interface (ALI) cultures (Fig. 2B). In primary CFBE cells transfected in submerged culture using LPRs formulated with the targeting peptide Y, the efficiency of silencing was 59.5% at a siRNA concentration of 50 nM and 72.9% at 100 nM (Fig. 3A) which was slightly lower than the silencing seen with LPRs formulated with peptide E (Fig. 2A). A decrease in knockdown efficiency mediated by peptide Y-LPRs was observed in CF cells grown at ALI (Fig. 3C), as shown for NHBE cells, with expression of the α-ENaC subunit transcripts reduced by 28.2% at 30 nM and 43.3% at 50 nM.

The effect of these formulations on CFBE cell viability in comparison to cells that were not transfected, ranged between 72% at 50 nM and 57% at 100 nM for α-ENaC-targeted siRNA, whereas the cell survival was higher at the same concentrations of non-targeted/irrelevant siRNA (Fig. 3B), although there was no statistical difference among the different groups.

### Biophysical properties and transfection efficiencies of nebulised LPRs

**Figure 4:**
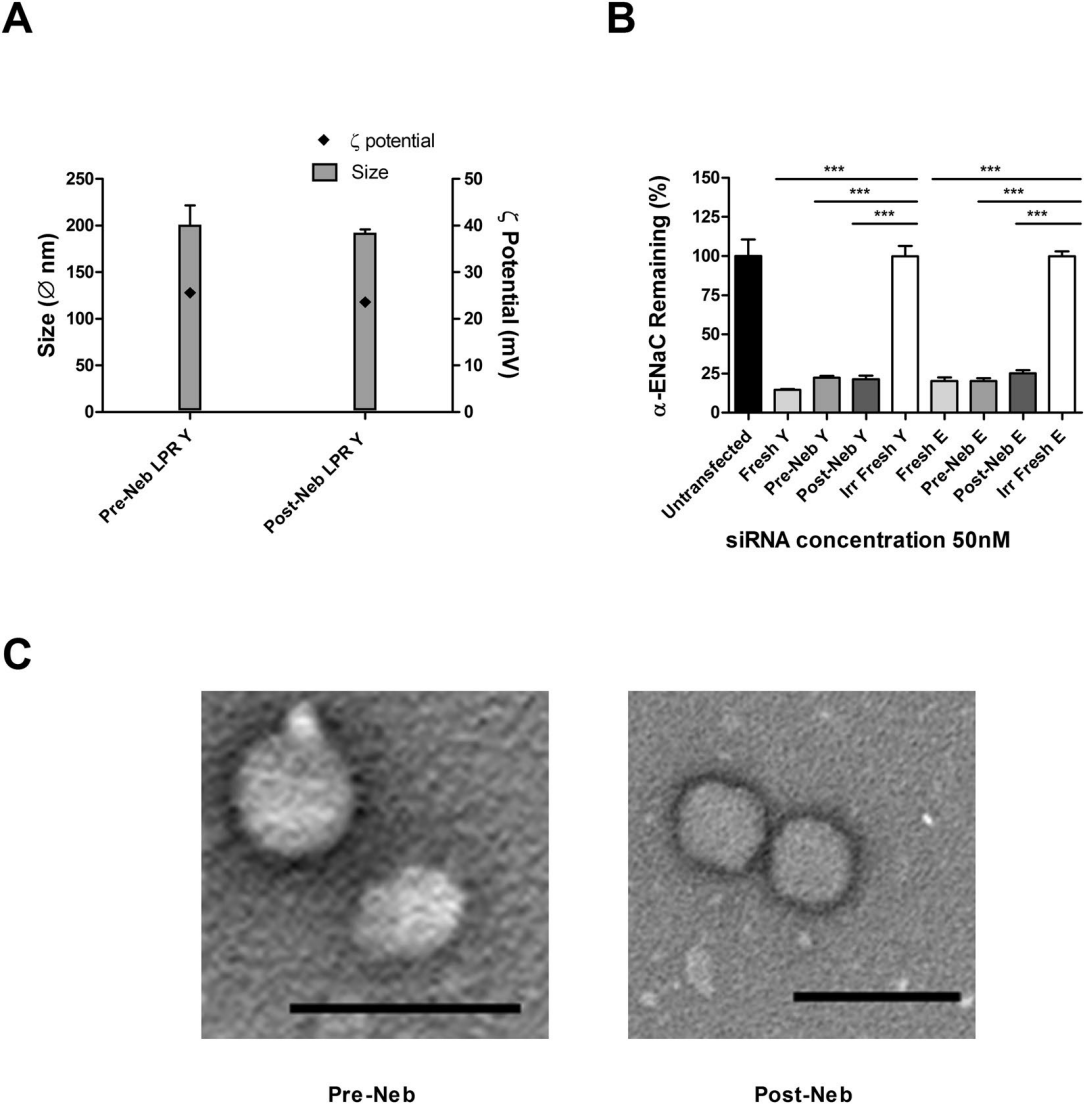
Nebulised LPRs. LPR particles were formulated at a 1:4:1 ratio of DOTMA/DOPE, peptide either Y or E and 100 μg of siRNA in 2 ml volume of H_2_O. (**A**) The particles sizes and the ζ potentials were assessed. The LPRs displayed sizes between 192 and 200 nm in diameter while the ζ potentials were between 23.6 and 25.6 mV. (**B**) The **s**ilencing capability of LPRs was detected after transfection of human airway epithelial (HAE) cells. The transfection ability of LPRs containing either peptide Y (n = 5) or peptide E (n = 6) and 50 nM siRNA was measured comparing the un-nebulised freshly made LPRs, the pre-nebulisation and post-nebulisation formulations relative to the freshly made non-targeting/irrelevant siRNA. Liposome-peptide Y- RNA nanoparticles freshly prepared or pre- and post-nebulisation displayed a silencing efficiency of 85.4%, 77.7% and 78.5%, respectively. Peptide E-LPRs showed similar levels of ENaC silencing 79.8% mediated by freshly prepared and pre-nebulisation, and 74.7% mediated by the nebulised LPRs. Statistically significant differences were determined as ***P ≤ 0.001. (**C**) LPRs pre- and post-nebulisation were also assessed by transmission electron microscopy (TEM). Size bars are 500 nm.

Aerosol delivery is an attractive approach for therapeutic interventions in the affected epithelial cells in the lungs of CF patients and we have previously shown that LPRs can deliver genes to the airway epithelium by nebulisation^19, 22^. LPRs were formulated in water, nebulised through an AeroEclipse II BAN jet nebuliser and the aerosol was collected. When loading the nebuliser with a small volume only a maximum of 50% of the suspension can be released as aerosol^23^, however, the amount of LPR suspensions nebulised here was 68.8 ± 4.7% (n = 3, Supplementary Table S1). In the same experiments the yield of siRNA in the collected nebulised LPRs, expressed as percentage of the siRNA in the pre-nebulisation suspension, was 17.5 ± 4.6%. Samples, prior to and after nebulisation, were stored refrigerated then investigated for biophysical properties and efficiency of silencing (Fig. 4A). The LPR sizes showed no significant variation after nebulisation at 200 ± 21 nm pre-nebulisation and 192 ± 4 nm after nebulisation. The ζ potential also displayed a similar colloidal stability, showing values of 25.6 ± 2.6 mV for the pre-nebulised LPRs versus 23.6 ± 0.4 mV for the aerosolised ones. Also TEM images showed that the LPR suspension pre- and post-nebulisation were homogeneous and quite similar (Fig. 4C).

In order to determine the ability of post-nebulised LPRs, formulated with either peptides (Y and E), to knockdown the transcript for the α-ENaC gene *in vitro*, equal amounts of freshly made LPRs, pre-nebulised nanoparticles suspension and nebulised material were added to cultures of 1HAEo− cells (Fig. 4B). The silencing efficiency of Y-LPRs was 77.7% for the pre-nebulisation suspension and 78.5% for the post-nebulisation formulation, compared to 85.4% for their freshly made counterparts. Similar α-ENaC mRNA knockdown efficiencies were shown by the E-LPRs; both the freshly made preparation and the pre-nebulisation formulation displayed a similar silencing ability of 79.8%, whereas the expression of α-ENaC showed a transcript knockdown of gene equal to 74.8% by the post-nebulisation suspension.

### Detection of α-ENaC protein by confocal microscopy and western blotting

**Figure 5:**
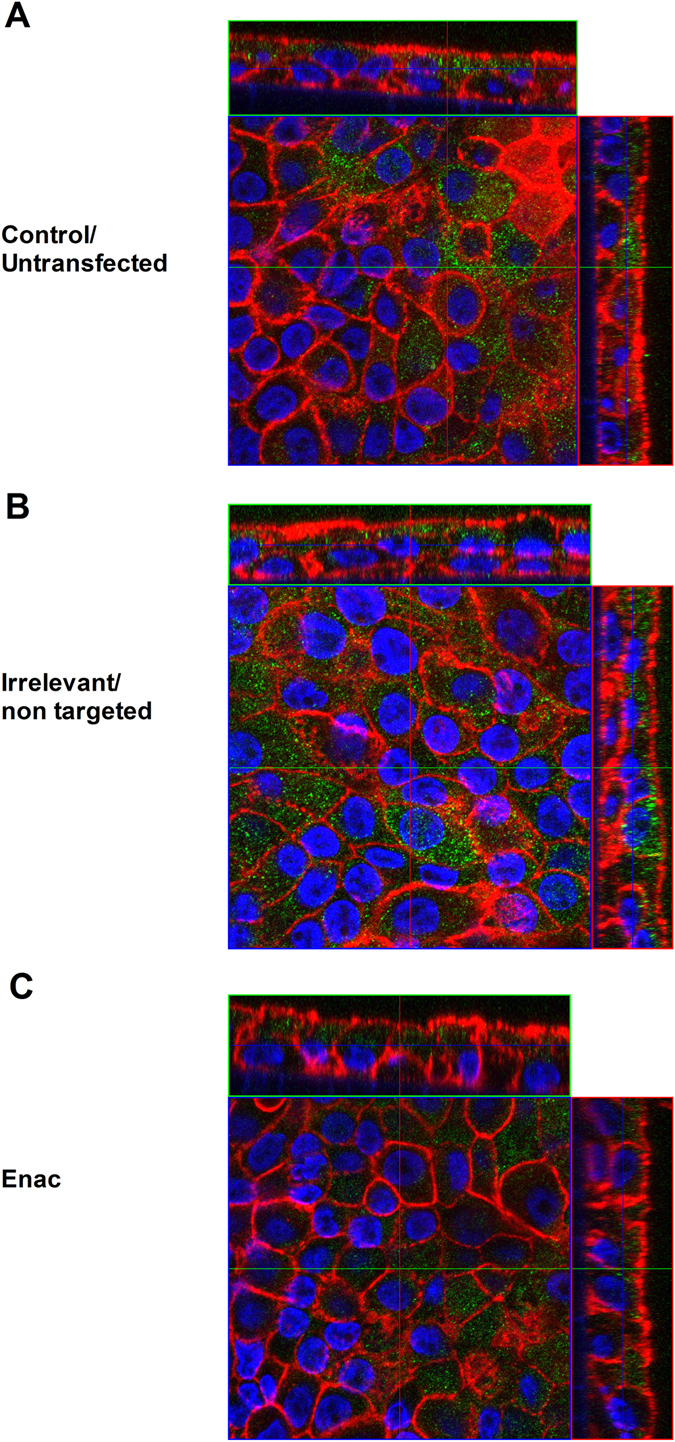
Confocal images of primary cells transfected with peptide E nanoparticles. Confocal images of NHBE cells grown in transwells and stained for α-ENaC (green), actin (red) and DAPI (blue) to label the nuclei; (**A**) untransfected control (**B**) non-targeting/irrelevant siRNA-treated and (**C**) α-ENaC siRNA-treated. The narrow panels on top and on the right side represent the cross-sectional view related to the indicated lines.

Confocal microscopy experiments were performed on NHBE cells grown in ALI culture (Fig. 5A–C), transfected with LPRs containing peptide E and siRNA at 50 nM concentration then stained for α-ENaC subunit (green), actin (red) and DAPI (blue). Although quantification was not possible due to the non-homogeneous growth of the cells in ALI cultures and the worsening of the spherical aberration when focusing in the depth of the transwell, a decrease in the fluorescent staining of the α-subunit was detected.

**Figure 6:**
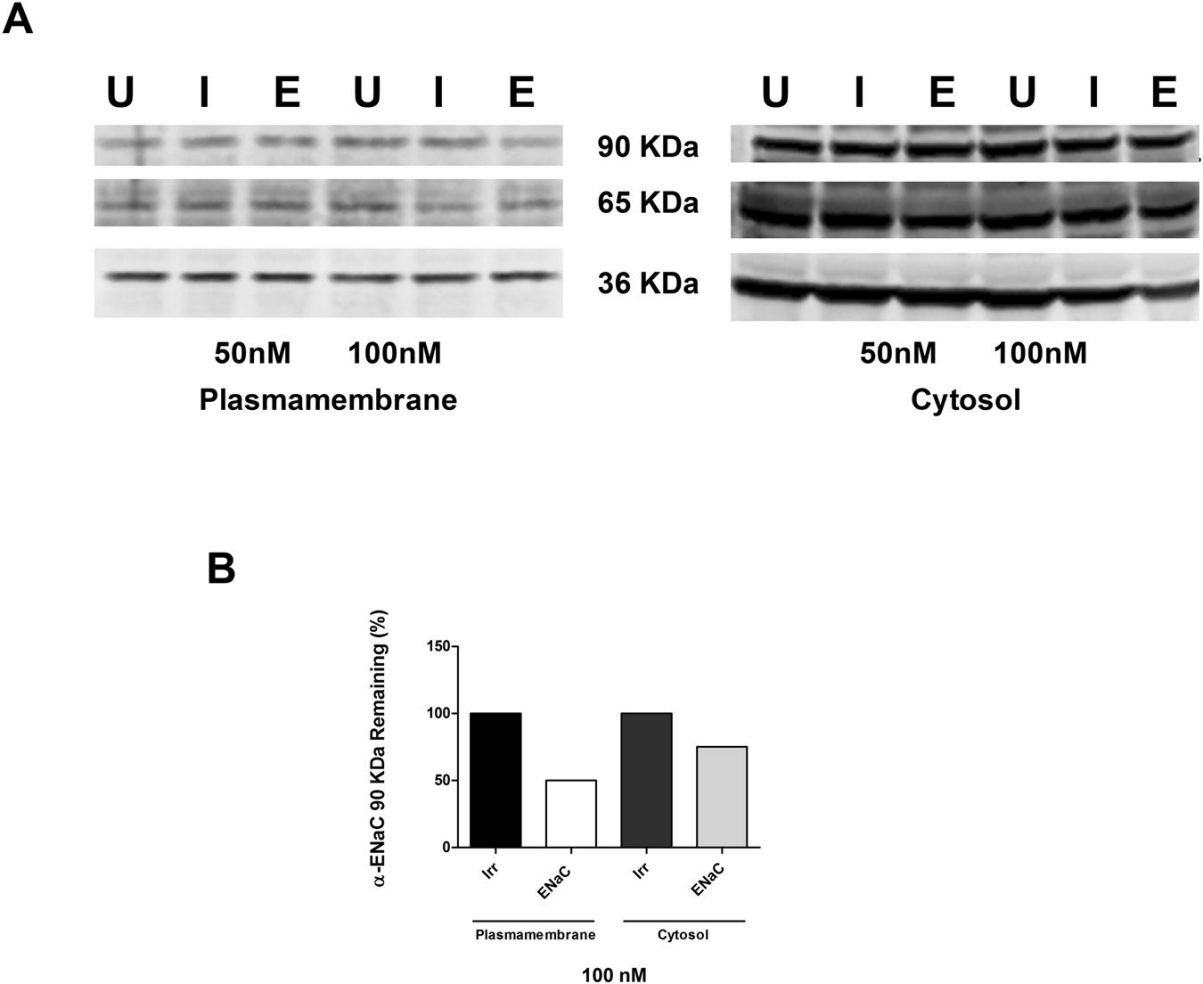
Western blotting of α-ENaC protein from A549 cells transfected with peptide E-LPRs. (**A**) Representative western blotting showing the α-ENaC major forms of 90 KDa and 65 KDa bands protein levels after treatment with LPR at both 50 nM and 100 nM siRNA, and GAPDH (36 KDa band). The lanes are indicated as following: untransfected control (U), treated with non-targeting/irrelevant siRNA (I) and α-ENaC siRNA-treated (E). (**B**) The densitometry results of 90 KDa α-ENaC protein from siRNA-treated cells extracted from the plasma membrane and from the cytosolic fraction, and expressed as percentage relative to the non-targeting/irrelevant siRNA-treated at a concentration of siRNA of 100 nM, showed that 50% of the subunit was still present at the cell surface, while 75% was still present in the cytosol.

The silencing of the protein was then investigated by western blotting assessing the amount of α-ENaC at the plasmalemma level and in the intracellular pool. Despite the observation that mRNA was efficiently silenced at a concentration of 50 nM siRNA, a decrease in the protein level of the 90 kDa α-subunit was seen only with 100 nM siRNA (Fig. 6A and B). A decrement was also seen in the 65 kDa band at least in the cytosolic fraction of ENaC siRNA treated cells compared to the irrelevant siRNA (Fig. 6A). Quantification of the 90 kDa bands that were detected on the western blots (Fig. 6B) showed that the percentage of knockdown normalised to the non-targeting siRNA was 50% and 25% in plasma membrane and cytosolic fractions, respectively.

### SiRNA delivery *in vivo*

**Figure 7:**
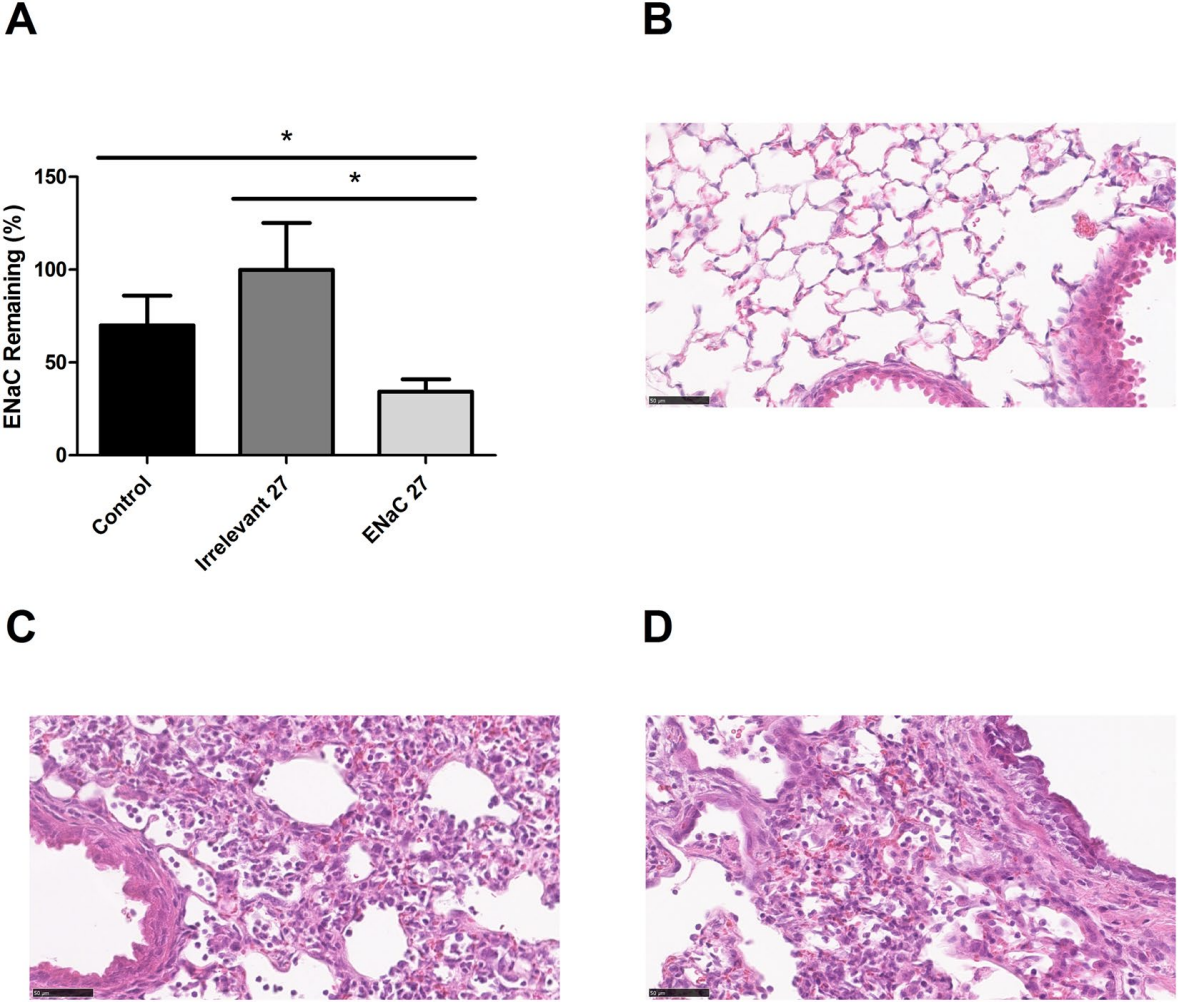
*In vivo* delivery of LPRs containing 27 μg siRNA. (**A**) Following the LPRs administration, the knockdown in the expression of the α-ENaC gene mRNA in the lungs of C57BL6 female mice was measured by qRT-PCR. β actin was used as reference gene for mouse lung transcripts. The level of transcript expression of α-ENaC siRNA-treated group was compared with the control groups either untreated or non-targeting/irrelevant siRNA treated cohorts. Statistical analysis was performed using Student’s *t*-test. Statistical significance is represented by *P < 0.05. (**B–D**) Representative images of H&E stained lung sections from untreated control (**B**), non-targeting/irrelevant siRNA-treated (**C**) and α-ENaC siRNA-treated (**D**) mice show a mild peribronchial mononuclear cell infiltration in siRNA-treated animals (**C**,**D**) compared with untreated controls (**B**). Scale bars = 50 µm.

We then evaluated the *in vivo* knockdown of ENaC α-subunit transcripts. C57BL6 mice were treated with LPRs containing peptide Y and 27 μg of either ENaC siRNA or irrelevant/non- targeted control siRNA by oropharyngeal administration and the expression of α-ENaC was examined 48 h later. A statistically significant reduction (*P < 0.05) using Student’s *t*-test in the expression of α-ENaC (Fig. 7A) was observed between the ENaC-treated group and the control groups (either treated with non-targeting/irrelevant siRNA or untreated). H&E staining of the lungs showed that LPR administration to the lungs of mice, containing either ENaC-targeting or non-targeting siRNAs, induced a mild peribronchial proteinaceous and predominantly mononuclear cell infiltrate compared with untreated controls (Fig. 7B–D).

## Discussion

Despite the remarkable personalised approaches brought to CF therapy by the CFTR modulators, gene therapy still offers a valid option for patients with mutation classes where these new therapeutics are lacking. SiRNA approaches to silence ENaC may also be a co-adjuvant therapy not only for CF, but to all those treatments aiming to restore appropriate airway surface liquid volume and mucus hydration by regulating sodium hyperabsorption. Gene therapy for CF, targeting fully differentiated airway epithelial cells, has to take into account the dynamic airway plasticity that requires repeated applications. We have previously shown that LPR nanoparticles can be used to target different cell-types^18, 21, 24–26^. The aim of this study was to establish whether we could reduce the Na^+^ reabsorption in the airway epithelial cells and *in vivo* in the mouse lungs by silencing the sodium channel ENaC.

ENaC is a complex target because the channel is formed of different subunits and because the α-subunit can be alternatively spliced, can undergo post-translational modifications and can be highly glycosylated and ubiquitinylated^27–29^. It is important also to consider that ENaC is differentially expressed in cells and tissues^8^ and that the amount of intracellular protein seen by confocal microscopy in some cells could be due to the constant replacement of the subunit due to the proteolytic cleavage as part of one of ENaC regulatory mechanisms^29–31^.

We showed here that the LPR formulation has the ability to knockdown the α-ENaC subunit at various degrees in different airway epithelial cells. Although primary cells are usually more difficult to transfect, in general we found that better silencing was achieved in these cells rather than in cell lines, where a knockdown level of 50% or better was achieved. Although silencing efficiencies were slightly lower when the same cells were maintained in ALI culture, a more physiologically-representative model of the airway respiratory epithelium, here the knockdown levels in CF primary epithelial cells were extremely encouraging, considering that ALI cultures are notoriously difficult to transfect. Interestingly, the amount of α-ENaC mRNA did not correspond to protein levels, which may be due to translational regulation of the mRNA, protein stability or the α-subunit forming a conducting channel with different regulatory characteristics. Our data also demonstrated that either targeting peptides were equally able to mediate the delivery of liposomal nanoparticles to the airway epithelial cells.

Consistent with our previous results^18^, the LPRs had the desired biophysical characteristics for a delivery vector in terms of size and stability. The biophysical analysis revealed that all the LPR formulations displayed sizes suitable for cellular uptake^24^ and showed enhanced colloidal stability, which render them less prone to aggregation or flocculation. LPR formulations made in water and nebulised by AeroEclipse II BAN jet nebuliser retained their biophysical properties and their ability to silence the α-ENaC gene in airway epithelial cells suggesting that LPRs presented in the current work may also be useful for clinical translation.

We further demonstrated that LPRs have the ability to knockdown the gene encoding the α-ENaC subunit in C57BL6 mouse lungs. The experiments showed that there was a certain degree of silencing of the gene in the ENaC siRNA-treated mice. A complete inactivation of the α-ENaC gene in mouse has been reported to abolish the Na^+^ transport in airway epithelia causing the development of respiratory distress due to the inability to clear the lung of liquid and death of the new-borns within 40 h from birth^32^. The degree and nature of mild oedema seen in the siRNA-treated mice is probably a non-specific effect of LPR administration.

Overall, our study demonstrates that LPRs can be used to effectively deliver siRNA to the airway epithelium and that the expression of α-ENaC can be modulated *in vitro* and *in vivo*. However, an optimal dose is required to achieve the therapeutic benefits without potential side effects. Receptor-targeted liposome-peptide-siRNA nanoparticles represent an efficient and safe non-viral siRNA delivery system that could be used to modulate sodium hyperabsorption, thus helping to restore the correct volume of surface liquid, mucus hydration and mucociliary clearance in the airways, in cystic fibrosis.

## Methods

### Cell lines, primary cells and air-liquid interface (ALI) cultures

The 16HBE14o- and 1HAEo− cells (kindly provided by Dieter Gruenert, California Pacific Medical Center Research Institute, San Francisco, CA, USA) were maintained in Minimum Essential Medium Eagle’s modification (Sigma, Poole, UK) at 37 °C in a humidified atmosphere with 5% CO_2_. Tissue culture medium was supplemented with 10% heat-inactivated foetal bovine serum (FBS, Life Technologies, Paisley, UK), 2 mM L-glutamine (Life Technologies) and 0.1 mM non-essential amino acids (Sigma). The adenocarcinoma human alveolar basal epithelial cell line, A549 (ATCC® CCL-185™), was cultured in Dulbecco’s Modified Eagle Medium (DMEM) - GlutaMAX™ (Life Technologies) containing 10% FBS. The H441 human lung adenocarcinoma epithelial cell line, commonly used as a model of club cells, was a kind gift from Guy Moss (UCL Neuroscience, Physiology & Pharmacology, London, UK). The H441 cells in submerged cultures were maintained in RPMI1640 ATCC Modification (Life Technologies), 10% FBS, 1% InsulinTransferinSelenium (Life Technologies), 1 mM Sodium Pyruvate (Life Technologies), 2 mM LGlutamine (Life Technologies) whereas the H441 ALI culture medium contained also 0.2 mM Dexamethasone (Sigma), 10 nM TriiodoLthyronine (Sigma) and 4% Charcoal Stripped Serum (Sigma) instead of the FBS mentioned above. Normal Human Bronchial Epithelial cells (NHBE) were obtained from Lonza (Lonza Biologics plc, Cambridge, UK) and the Cystic Fibrosis Bronchial Epithelial cells (CFBE) were obtained from Epithelix (Epithelix SàRL, Genève, Switzerland). Both primary cells were transduced with a lentiviral vector expressing the anti-senescent *BMI*-*1* oncogene^33, 34^. Primary cells in submerged cultures were grown on 1% collagen-coated plastic flasks (PureCol® Bovine Collagen Solution, Type I, Advanced BioMatrix, San Diego, CA, USA) in Bronchial Epithelial Growth Medium (BEGM, Lonza) supplemented with Bovine Pituitary Extract, hydrocortisone, Human Epidermal Growth Factor, epinephrine, transferrin and insulin.

For ALI culture, primary cells were grown in 12 mm collagen-coated transwell inserts (Polyester (PET) Membrane Transwell-Clear Inserts, Corning, Corning Inc. Life Sciences, Tewksbury, MA, USA) at a seeding density of 1.5 × 10^5^ or 1 × 10^6^ viable cells per insert and fed with BEGM ALI medium (1:1 DMEM-Hi glucose: BEGM containing supplements) supplemented with 100 nM retinoic acid (Sigma). For the first two days, the apical side of the insert contained 250 μl of BEGM media and the basolateral side 750 μl of the same medium, then the medium from the apical side was removed and the basolateral replaced with the same volume (750 μl) of BEGM ALI medium.

### Liposome-Peptide-RNA complex formulation and biophysical analysis

Liposomes were purchased from Avanti Polar Lipids Inc. (Alabaster, AL, USA) while peptides were synthesized by Zinsser Analytic (Maidenhead, UK). Non-targeting siRNA was obtained from Thermo Fisher Scientific Inc. (Warrington, UK) and from Life Technologies (Paisley, UK). The α-ENaC subunit siRNA sequence was selected from an initial comparison of four candidate siRNAs in transfections of 16HBE14o- (5′-GCAGUGAUGUUCCUGUUGA-3′). This was obtained from Thermo Fisher Scientific Inc. (Warrington, UK) for *in vitro* transfections. The silencer® pre-designed siRNA for *in vivo* transfections was from Ambion (gene id: Scnn1a - AM16830 – Life Technologies, Paisley, UK). Cationic Lipid-Peptide-RNA (LPR) nanoparticles were formulated in OptiMEM (Life Technology, Paisley, UK) for *in vitro* experiments, at a weight ratio of 1:4:1 (liposome:peptide:siRNA), by adding the peptide E (K_16_GACSERSMNFCG) or Y (K_16_GACYGLPHKFCG) to the DOTMA/DOPE liposome and then mixed quickly with the siRNA usually to 50 or 100 nM final concentration, unless stated otherwise. Complexes were then left to assemble for at least 30 min up to 1 h at r.t. before being used for transfection experiments.

Cells grown in wells of plates or transwells were incubated with LPRs in OptiMEM for 4 h at 37 °C. The medium containing the nanoparticles was then replaced by fresh growth medium and the cells were incubated for a further 48 h prior to RNA extraction to measure the silencing efficiency by qRT-PCR, or lysed to detect the knockdown of the protein by western blotting or rinsed with PBS and fixed for confocal microscopy.

Aliquots of the LPR nanocomplexes were prepared and diluted in H_2_O (Life Technology) to a final volume of 500 µl. Complex sizes and ζ potentials were measured by dynamic light scattering and by laser Doppler anemometry using a Nano ZS Zetasizer (Malvern Instruments, Malvern, UK) with the settings described previously^19^. A total of three measurements per sample were taken automatically. For TEM analysis, the LPR nanocomplexes were prepared in water and applied onto a glow-discharged 300-mesh copper grid coated with a Formvar/carbon support film (Agar Scientific Ltd, Stansted, UK). After a few seconds, the grid was dried by blotting with filter paper. The sample was then negatively stained with 1% (w/v) uranyl acetate for a few seconds, before blotting with filter paper and air-dried. Imaging was carried out under a Philips CM120 BioTwin Transmission Electron Microscope and operated at an accelerating voltage of 120 KV.

### Cell Viability

Cell viability was assessed by measuring the release of lactate dehydrogenase (LDH) from damaged cells with the CytoTox-ONE Homogeneous Membrane Integrity Assay kit (Promega, Southampton, UK). Seeded cells were transfected with LPRs as above, then the culture supernatant was collected and mixed with an equal volume of the CytoTox-ONE reagent, according to the manufacturer’s instructions. The fluorescence (excitation λ 560 nm – emission λ 610 nm) was measured on a FLUOstar Optima spectrophotometer (BMG Labtech, Aylesbury, UK). Cell viability for each complex was expressed as a percentage of the viability of untreated cells.

### LPR nebulisation

For the nebulisation studies, 2 ml of LPR nanoparticles containing 100 μg of siRNA, prepared in H_2_O (Life Technologies), were added to the sample chamber of the AeroEclipse II BAN nebuliser, a kind gift from Trudell Medical International Europe Ltd (Manchester, UK). The nebulizer was then connected to the compressor Hikoneb® AeroCare II (Piston Type Nebuliser, Antek Electronic Ltd, Ankara, Turkey). The mouthpiece of the nebuliser was modified and connected by an adaptor to a large semi-rigid plastic tube (about 15 cm long) inserted two-thirds of its length inside a 50 ml tube through its cap, so that the aerosolised material would remain in the bottom. Also in the cap a barrel of a luer lock 1 ml syringe was inserted upside-down and attached to a tubing that helped to direct the flow out of the barrel into the water (placed in a Erlenmeyer flask), but could also allow some of the condensed material to fall back into the tube. The tube was pre-chilled and kept on ice to minimise evaporation. The nebulisation was performed for 15 min with the AeroEclipse set on continuous operational mode. Pre- and post-nebulisation samples were collected with a precision pipette and stored at 4 °C until required for further analysis. The concentrations of siRNA in LPRs pre-nebulisation, post-nebulisation and also from the left-over material in the sample chamber of the nebuliser were measured using Nanodrop 1000 (Thermo Fisher Scientific Inc., Wilmington, USA). The biophysical properties were then measured and their silencing activities were analysed in cellular transfections added at a concentration of 50 nM per well.

### RNA extraction from cells and mouse tissues

Lysates from cells washed in PBS, scraped and frozen at −80 °C until use, were first homogenized with Precellys (2 cycles × 5600 rpm, 30 sec per cycle) and then mixed with 1 vol of TRIreagent (Sigma). Mice lungs in 1–1.4 mL of TRIreagent (Sigma) were directly homogenized with the Precellys (4 cycles × 5600 rpm, 30 sec per cycle). The homogenates were centrifuged at 1000 g for 10 min at 4 °C and the supernatants were collected. After 5 min 1/5 vol of chloroform was added, following 3 min incubation the samples were centrifuged at 12000 g for 5 min at 4 °C. The supernatants were collected, added to an equal volume of 70% ethanol and further purified using the ISOLATE II RNA Micro Kit (Bioline, London, UK) according the manufacturer’s instructions. Samples were frozen at −80 °C until required for analysis.

### Reverse transcription and Real-time PCR

Total RNA was defrosted, treated with DNase (1 μg) and either converted into first-strand cDNA using random hexamers and MuLV reverse transcriptase (Life Technologies) for 1 h at 42 °C and cDNA was subsequently heated at 80 °C for 5 min prior to qRT-PCR assay or used in a one-step qRT-PCR (SensiFAST™ Probe Hi-ROX Kit, Bioline) combining the reverse transcription reaction with the qPCR reaction. Human or mouse ENaC, β-actin or GAPDH were quantified by Taqman primers and probes (Hs01013028_m1, Hs99999903_m1 and Hs03929097_g1, or Mm01182998_g1, Mm00607939_s1 and Mm99999915_g1, all from Life Technologies). The qRT-PCR assays were performed in an ABI PRISM® 7000 with the following default PCR parameters set at 50 °C for 2 min, 95 °C for 10 min and then 40 cycles at: 95 °C for 15 s and 60 °C for 1 min. The default parameters for one-step qRT-PCR were: 45 °C for 20 min, 95 °C for 2 min and then 40 cycles at: 95 °C for 15 s and 60 °C for 1 min. Only for A549 cells PrimePCR™ Probe Assays (Bio-Rad Laboratories Ltd, Hemel Hempstead, UK) were used: Human SCNN1A (qHsaCEP0024081 FAM) and Human GAPDH (glyceraldehyde-3-phosphate dehydrogenase, qHsaCEP0041396 Hex). GAPDH mRNA, in comparison with β actin, seemed to be a more suitable reference gene from *in vitro* transfections because showed minimal changes in expression levels between the individual samples and experimental conditions.

### Western blotting

Proteins from cells and ALI cultures were isolated and separated into a cytosolic and a membrane fraction using the ProteoExtract Transmembrane Protein Extraction Kit (Millipore Ltd, Watford, UK) according to the manufacturer’s instruction. The proteins were then concentrated by centrifugation at 12000 g for 30 min using Amicon Ultra-0.5 mL Centrifugal Filters (Millipore UK Ltd). For western blotting, the concentration of protein in the lysates was first measured by Pierce™ BCA Protein Assay (Pierce Biotechnology, Rockford, lL, USA) reading the absorbance at 595 nm in a FLUOstar Optima luminometer (BMG Labtech, Aylesbury, UK). Equal amounts of protein were then added into 4–12% NuPAGE® bis-tris SDS-PAGE gels (Life Technologies, Paisley, UK) and the gels were run for 35 min with MOPS buffer. Proteins were then wet-transferred onto nitrocellulose (Ø45 µm, Schleicher & Schuell Protran™ BA85 Nitrocellulose Membranes, Sigma). Membranes were left for 1 h in TBS-1% semi-skimmed milk then washed twice with Tris-buffered saline/Tween (TBST). The membrane was then probed overnight with a custom anti-α-ENaC antibody diluted 1:500 in 1% semi-skimmed milk/TBST. The custom anti-α-ENaC antibody, generated by Cambridge Research Biochemicals, was a kind gift of Ken Clark, Therapeutic Oligo DPU, Glaxo SmithKline, Stevenage, UK. After washing in TBST, the membrane was incubated for 1 h at room temperature with the goat anti-rabbit IgG conjugated with horseradish peroxidase (Life Technologies, Paisley, UK) diluted 1:10,000 in TBST. Following further washing the blot was developed with SuperSignal West Pico Chemiluminescent Substrate (Thermo Fisher Scientific Ltd, Cramlington, UK). The western blot densities, following the rolling ball background subtraction set at 50, were analysed using ImageJ programme (NIH, Bethesda, MD, USA, NIH.gov).

After stripping with Restore™ western blot stripping buffer (Thermo Fisher Scientific -Pierce), GAPDH bands were detected by probing the membranes overnight with a mouse anti-GAPDH antibody (Santa Cruz Biotechnology, Inc., Heidelberg, Germany) 1:500 in TBST, followed by a horseradish peroxidase-labelled rabbit polyclonal anti-mouse immunoglobulins (1:1000 in TBST; Dako UK Ltd, Ely, UK).

### Confocal Microscopy

Primary cells were grown in ALI cultures and transfected as above. Transwell inserts were then rinsed with PBS and fixed with 4% paraformaldehyde, washed with PBS, permeabilised with PBS containing 0.1% Triton for 10 min and blocked for 1 h with 3% BSA in PBS containing 0.1% Triton. The transwell membranes were stained with anti-α-ENaC rabbit polyclonal IgG antibody (Thermo Scientific) diluted 1:100 in 2% BSA and 0.03% Triton in PBS for 2 h. Transwell membranes were then washed thoroughly and incubated with Alexa Fluor 468 Phalloidin (Life Technologies) to stain F-actin diluted 1:100 and with goat anti-rabbit IgG conjugated with Alexa Fluor 488 (Life Technologies) diluted 1:1000 in 1% BSA and 0.03% Triton in PBS for 1 h. The inserts were finally washed with PBS and with distilled water prior adding the mounting medium (VectaShield, Vector Laboratories Ltd, Peterborough, UK) containing DAPI to stain the nucleus. The inserts were then mounted onto slides and kept in the dark at 4 °C. Images were acquired using a Zeiss LSM 710 confocal microscope (equipped with 7 laser lines: 405, 458, 488, 514, 561, 594 and 633 nm, and with 63x Plan-Apochromat NA 1.4 Oil objective) and processed using ImageJ software (NIH.org).

### Oropharyngeal delivery and histological staining of the lungs

For *in vivo* studies, 55 μl of LPR suspensions containing 27 μg ENaC targeting or irrelevant siRNA were administered through the oropharynx of 6–8 weeks old C57BL6 female mice (Charles River, Margate, UK) as described [35]. The third group of untreated mice was used as a control. Animals were killed 48 hours later and the lungs either excised and quickly frozen in liquid nitrogen for mRNA expression analysis as described above or alternatively the lungs were inflation fixed *in situ* via cannulation of the trachea with 4% paraformaldehyde at a pressure of 20 cm for histological analysis. Following inflation fixing, the lungs were excised, dehydrated and processed to paraffin wax as described previously^36^. Histological sections were dewaxed, rehydrated, and stained with haematoxylin and eosin as described previously^22^. All animals were handled in strict accordance with UCL animal care policies and all the procedures were carried out in accordance with the United Kingdom Animals (Scientific Procedures) Act 1986 (UK) and E.U. regulations. The work was approved and carried out under Home Office Project Licenses 70/6149 and 70/7073.

### Statistical Analysis

All graphs display mean and standard error of the mean (SEM). Statistical analysis was performed using the Student’s *t*-test to calculate statistically significant differences and individual P values using GraphPad Prism version 5.00 for Windows, GraphPad Software, San Diego California, USA, www.graphpad.com.

Statistically significant differences were expressed as *P ≤ 0.05, **P ≤ 0.01 and ***P ≤ 0.001, Not statistically significant differences (P > 0.05) are not shown.

## Electronic supplementary material

Supplementary Table S1
