# Supplementary material for: Delivery of ENaC siRNA to epithelial cells mediated by a targeted nanocomplex: a therapeutic strategy for cystic fibrosis
Source: Sci Rep. 2017 Apr 6;7:700. doi: 10.1038/s41598-017-00662-2 (PMC5428798; doi:10.1038/s41598-017-00662-2)
Supplement: Supplementary file 1 — Supplementary Table S1 [file 41598_2017_662_MOESM1_ESM.docx]

**SUPPLEMENTARY TABLE**

**Delivery of ENaC siRNA to epithelial cells mediated by a targeted nanocomplex: a therapeutic strategy for cystic fibrosis**

Maria D. I. Manunta^1^, Aristides D. Tagalakis^1^, Martin Attwood^1,3^, Ahmad M. Aldossary^1^, Josephine L. Barnes^2^, Mustafa M. Munye^1^, Alexander Weng^1,4^, Robin J McAnulty^2^, and Stephen L. Hart^1^

**^1^Experimental and Personalised Medicine Section, UCL Great Ormond Street Institute of Child Health, London, UK;** ^2^UCL Respiratory Centre for Inflammation and Tissue Repair, University College London, London, UK; ^3^current address: Centre for Cellular and Molecular Physiology, University of Oxford, Oxford, UK; ^4^current address: Institute of Pharmacy, Freie Universität Berlin, Berlin, Germany.

| **Nebulisation output** | | | | | | | | | |
| --- | --- | --- | --- | --- | --- | --- | --- | --- | --- |
|  | Pre - Neb | | Post - Neb | | | Left-Over | | |  |
|  | Conc  (μg/ml) | Vol  (ml) | Conc  (μg/ml) | Vol  (ml) | Yield (%) | Conc  (μg/ml) | Vol  (ml) | Yield (%) | Mass emitted (%) |
| # 1 | 50 | 2 | 20.1 | 1.102 | 22.2 | 61.8 | 0.424 | 26.2 | 73.8 |
| # 2 | 50 | 2 | 10.4 | 1.237 | 12.9 | 70.3 | 0.456 | 32.1 | 67.9 |
| # 3 | 50 | 2 | 16.8 | 1.046 | 17.6 | 70.8 | 0.500 | 35.4 | 64.6 |
| Mean ± SD | - | - | 15.8 ± 4.9 | 1.128 ± 0.1 | 17.5 ± 4.6 | 67.6 ± 5.1 | 0.460 ± 0.0 | 31.2 ± 4.7 | 68.8 ± 4.7 |

**Supplementary Table S1:** **Summary of the nebulisation output of LPRs from 3 experiments.** Left–over is the amount of LPR suspension remaining in the nebuliser chamber after nebulisation. Yield (%) is calculated from the amount (μg) of siRNA recovered after nebulisation versus the amount (μg) that was applied to the nebuliser, which is set at 100%. Mass emitted is the percentage of the siRNA in the LPR suspensions that was released as aerosol. Conc = concentration of the siRNA in LPRs: Pre-Neb, Post-Neb and in the Left-Over solutions; Vol = volume of a) Pre-Neb material that was nebulised, b) Post-Neb material collected from the receiver tube and c) Left-Over material that was not nebulised and collected from the sample chamber of the nebuliser.
